# Large-scale SNP screenings identify markers linked with GCRV resistant traits through transcriptomes of individuals and cell lines in *Ctenopharyngodon idella*

**DOI:** 10.1038/s41598-017-01338-7

**Published:** 2017-04-26

**Authors:** Zhiwei Liao, Quanyuan Wan, Xueying Shang, Jianguo Su

**Affiliations:** College of Fisheries, Huazhong Agricultural University, Wuhan, 430070 China

## Abstract

Grass carp (*Ctenopharyngodon idella*) is an important economic species in freshwater aquaculture and its industry has been confined due to variety degeneration and frequent diseases. Marker-assisted selection is a feasible method for selective breeding of new varieties. Transcriptome data have greatly facilitated high-throughput single nucleotide polymorphism (SNP) marker discovery and phenotype association study. In this study, we gained a total of 25,981 and 5,775 high quality SNPs in two transcriptomes from individuals and cell lines, respectively. Comparative transcriptome analysis identified 413 and 832 grass carp reovirus (GCRV)-resistant-association SNPs as well as 1,381 and 1,606 GCRV-susceptible-association SNPs in individuals and cell lines, respectively. Integrated analysis indicated 22 genes with single SNP share common resistant/susceptible traits in two transcriptomes. Furthermore, we infected grass carp with GCRV, genotyping and association analyses were performed, and 9 in 22 SNPs were confirmed by PCR-RFLP. Meanwhile, mRNA expression profiles of 6 genes containing confirmed SNPs were examined by qRT-PCR. The results demonstrated that mRNA expressions were significant differences in resistant/susceptible individuals and cell lines. The present study develops an important strategy for high throughput screening of phenotype association genetic markers and the results will serve in grass carp breeding for GCRV resistance.

## Introduction

Grass carp (*Ctenopharyngodon idella*) is an important economic freshwater fish in China and accounted for 15.6% of global freshwater aquaculture production in 2011^1^. However, frequent diseases and growth degradation have restricted its development in aquaculture^2, 3^. Grass carp reovirus (GCRV) is a disastrous pathogen causing hemorrhagic disease, mainly infecting young fingerlings and yearlings of *C. idella* and black carp (*Mylopharyngodon piceus*)^2, 4^. This virus is widespread in south China and results in severe economic losses to aquaculture industry^5^. Numerous researches devote to find an effective approach to prevent this disease, i.e. drug screening, vaccine and RNAi^6^. However, comparing with those therapies, molecular breeding may be more environmentally friendly and sustainable. Inspiringly, the success of marker-assisted breeding (MAB) of a lymphocystis disease-resistant Japanese flounder (*Paralichthys olivaceus*) presents the prospect of anti-disease breeding^7^. As the primary task of MAB is to put plenty of genetic markers of immunity-associated genes on records^8^. Since the 1960s, various intra- and interspecific hybridization have been carried out and progresses have been made in the breeding of disease resistance in *C. idella*. As an example, specimens selected from a population in Heilongjiang River in China increase the GCRV resistance by 54.7%, which are harvested from the hybrid offspring of common carp (*Cyprinus carpio*) and *C. idella*, but has not been commercialized due to the genetic instability of hybrid offspring^2^. In summary, due to the long sexual maturation period (4–5 years) of *C. idella*, the traditional breeding method is inefficient and this is a common problem in fish breeding. Thus, it is crucial to improve the breeding efficiency to promote the cultivation of fish varieties.

Genetic single nucleotide polymorphisms (SNPs) in innate and adaptive immunity have aroused much attention and a number of studies have been conducted to identify SNPs in the genomes of diverse species^9^. SNPs are the most abundant type of DNA sequence polymorphisms whose applications have been proved in genetic studies^10^. They have been applied in quantitative trait loci (QTL) mapping and genome-wide association studies (GWAS) in model organisms and humans^11, 12^. In aquaculture species, SNP markers are becoming the important genetic resources in linkage map construction and association studies. In recent years, more efforts have been made for SNP discovery in fish^13, 14^. However, the number of SNPs is still insufficient for high density SNP chip construction and GWAS.

Next-generation sequencing (NGS) technologies have made high-throughput SNP discovery feasible for non-model species^13, 14^. Recently, transcriptome sequencing has become an important method for SNP discovery^15^. Through transcriptome sequencing, functional genes can be sequenced at high coverage, which ensures full-scale SNP discovery in protein-coding genes with high accuracy. Massive SNPs have been identified by transcriptome sequencing in aquaculture species such as catfish (*Clarias gariepinus*), Atlantic cod (*Gadus morhua*), oyster (*Crassostrea gigas*), shrimp (*Litopenaeus vannamei*), half-smooth tongue sole (*Cynoglossus semilaevis*), Atlantic herring (*Clupea harengus*), Atlantic salmon (*Salmo salar*), silver carp (*Hypophthalmichthys molitrix*) and *C. carpio* ^16^. These data supply a large amount of genetic information related to development and disease resistance in aquaculture species. However, SNP discovery associated with resistance/susceptibility to virus has not been reported in the transcriptome of fish.

SNPs applied in MAB is a potent method for selective breeding of disease resistance varieties. Herein, the transcriptomes of individuals and cell lines were employed to identify SNPs that involved in resistance/susceptibility to GCRV. Two transcriptomes were integrated to look for their communal variation locus to improve the SNPs accuracy, additionally, the candidate SNPs were confirmed by polymerase chain reaction-restriction fragment length polymorphism (PCR-RFLP) experiments. Furthermore, genotyping and association analyses were conducted to study the relationship between SNPs and antiviral activity by an independent infection experiment. Meanwhile, mRNA expression profiles of genes corresponding to confirmed SNPs were examined by quantitative real-time RT-PCR (qRT-PCR) as well as transcriptome data analysis in resistant and susceptible groups. These results provide precious resources for molecular and genetic breeding as well as immune researches.

## Results

### SNP detection and screening

**Figure 1:**
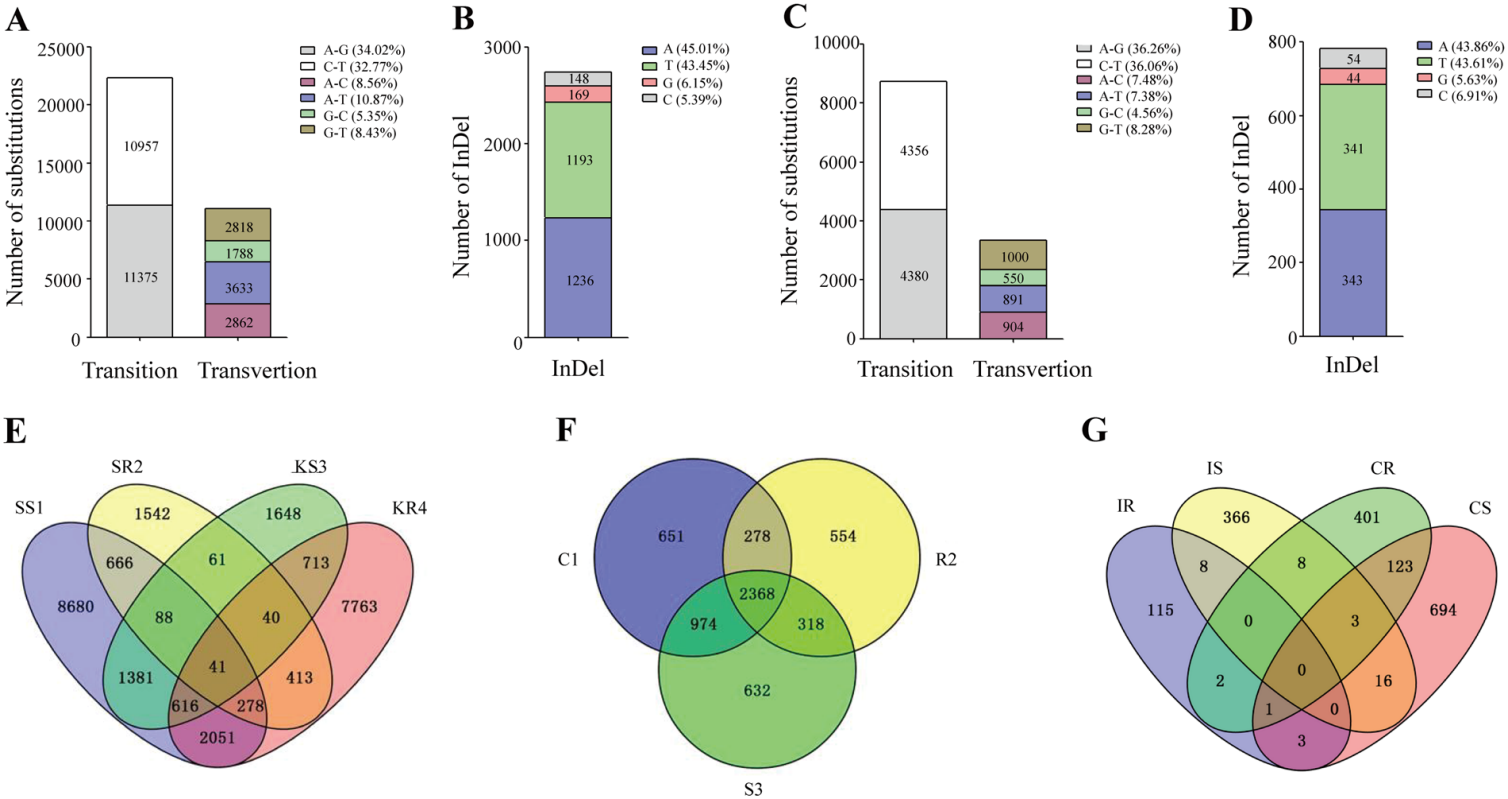
Statistical analyses and venn diagrams of SNPs, InDels and genes based on transcriptome datasets. (**A**) and (**B**) represent the substitutions and InDels in individuals, respectively. (**C**) and (**D**) represent the substitutions and InDels in cell lines, respectively. (**E**) Venn diagram describes the overlapped resistance/susceptibility-associated SNPs among SS1, SR2, KS3 and KR4 in individuals. (**F**) C1 represents SNPs from the transcriptome of control CIK cells, and R2 and S3 stands for SNPs in resistant and susceptible cell lines, respectively. (**G**) IR and IS represent genes containing resistance/susceptibility-associated SNPs in individual transcriptomes, respectively, CR and CS stand for genes containing resistance/susceptibility-associated SNPs in cell transcriptomes, respectively.

Illumina MiSeq (2 × 250 bp) in individuals and Illumina HiSeq2500 (2 × 125 bp) in cell lines generated 20.04 Gb (89.07% of raw data) and 17.23 Gb (96.74% of raw data) clean data bulk, respectively (Table 1). SAMtools were employed for SNP detection^17^. In the present study, SNP only refers to single base substitution (transition and transversion), InDel represents single base insertion and deletion. Meanwhile, in order to improve the accuracy of SNP analysis, those with total read depth over 20 and mutation read depth over 10 are regarded as high quality SNPs and InDels. A total of 33,433 and 12,081 high quality SNPs were picked out from SS1, SR2, KS3 and KR4 libraries of individuals and C1, R2 and S3 libraries of cell lines, respectively (Fig. 1A,C and Supplementary Dataset 1). In addition, a total of 2,746 and 782 high quality InDels were found in two transcriptomes, respectively (Fig. 1B,D and Supplementary Dataset 2). However, in view of the identical SNPs in SS1, SR2, KS3 and KR4 libraries, it showed a total of 25,981 SNPs in individuals. Similarly, taking into consideration the identical SNPs in C1, R2 and S3 libraries, it showed a total of 5,775 SNPs in cell lines (Fig. 1E and F). In the identified SNPs, the frequencies of transitions (66.79% in individuals and 72.32% in cell lines, respectively) were higher than those of transversions (33.21% in individuals and 27.68% in cell lines, respectively). In terms of transition, the similar amounts of A/G (11,375) and C/T (10,957) were found. Likewise, the frequencies of the four transversion types (A/C, A/T, G/C and G/T) were approximately alike as well (Fig. 1A and C). As expected, the ratio of transition to transversion was about 2.0. With respect to InDels, the amounts of A and T as well as G and C were similar, while the ratio of A and T to G and C was about 7.0 (Fig. 1B and D).

**Table 1:** Summary of the transcriptomes of *C. idella* in individuals and cell lines.

Type		Raw base pairs (bp)	Clean base pairs (bp)	≥Q20	Raw reads	Clean reads	≥Q20
**Individual**	Spleen (SS1)	7095838055	6627953846	93.41	32859452	32535864	99.02
	Spleen (SR2)	4215899019	3374188672	80.03	24450204	22841432	93.42
	Kidney (KS3)	5219216274	4406813074	84.43	19492284	17678754	90.70
	Kidney (KR4)	7631346510	7112896707	93.21	35183620	34903598	99.20
**Cell**	Control (C1)	6139459750	5930031750	96.59	49115678	47440254	96.59
	Resistant (R2)	6556782500	6341051750	96.71	52454260	50728414	96.71
	Susceptible (S3)	6427464500	6234584000	97.00	51419716	49876672	97.00

Note: Paired-end reads were generated in lengths of 2 × 250 bp and 2 × 125 bp in individuals and CIK cells by Illumina MiSeq and HiSeq2500, respectively. SS1 and SR2 represent susceptible and resistant spleen tissues, respectively. KS3 and KR4 represent susceptible and resistant head-kidney tissues, respectively. C1, R2 and S3 represent control, resistant and susceptible groups in cells, respectively. Q20: percentage is the proportion of nucleotides with a quality value ≥20 in reads.

Based on these high quality SNPs, exploring specific SNPs associated with resistance/susceptibility to GCRV was feasible. We used the venn diagram to display the SNPs among different tissues and cell lines by comparative transcriptome analysis. The results showed 1,381 identical SNPs between SS1 and KS3 (excluding SR2 and KR4) and 413 identical SNPs between SR2 and KR4 (excluding SS1 and KS3) in individuals, 832 specific SNPs in R2 (excluding S3) and 1,606 specific SNPs in S3 (excluding R2) in cell lines (Fig. 1E,F and Supplementary Dataset 3). In the subsequent analysis, these SNPs were regarded as resistant or susceptible SNPs in individuals and cell lines. Furthermore, in order to find communal SNPs in individuals and cell lines, these SNPs (1,381 and 413 in individuals, 832 and 1,606 in cell lines) were mapped to the corresponding genes. The results showed that 3 communal genes were shared by resistant individuals (IR) and cells (CR) as well as 19 communal genes were shared by susceptible individuals (IS) and cells (CS) (Fig. 1G). Meanwhile, These 22 genes possess single SNP associated with resistance/susceptibility to GCRV in individuals or cells, whose non-redundant annotations and gene names were listed in Supplementary Table S1. And the positions of SNPs in 22 genes except *C7N1* were different between individuals and cell lines, so there were totally 43 SNPs in the 22 genes in individuals and cells. To study the practical significance of SNPs, 22 SNPs in the transcriptome of individuals were selected for verification and association analysis, in which fish from different population were used. Meanwhile, other SNPs that were not selected for further analysis and verification might also play some roles in the antiviral immune responses.

### Read depth and distribution of SNPs associated with resistance/susceptibility to GCRV

**Figure 2:**
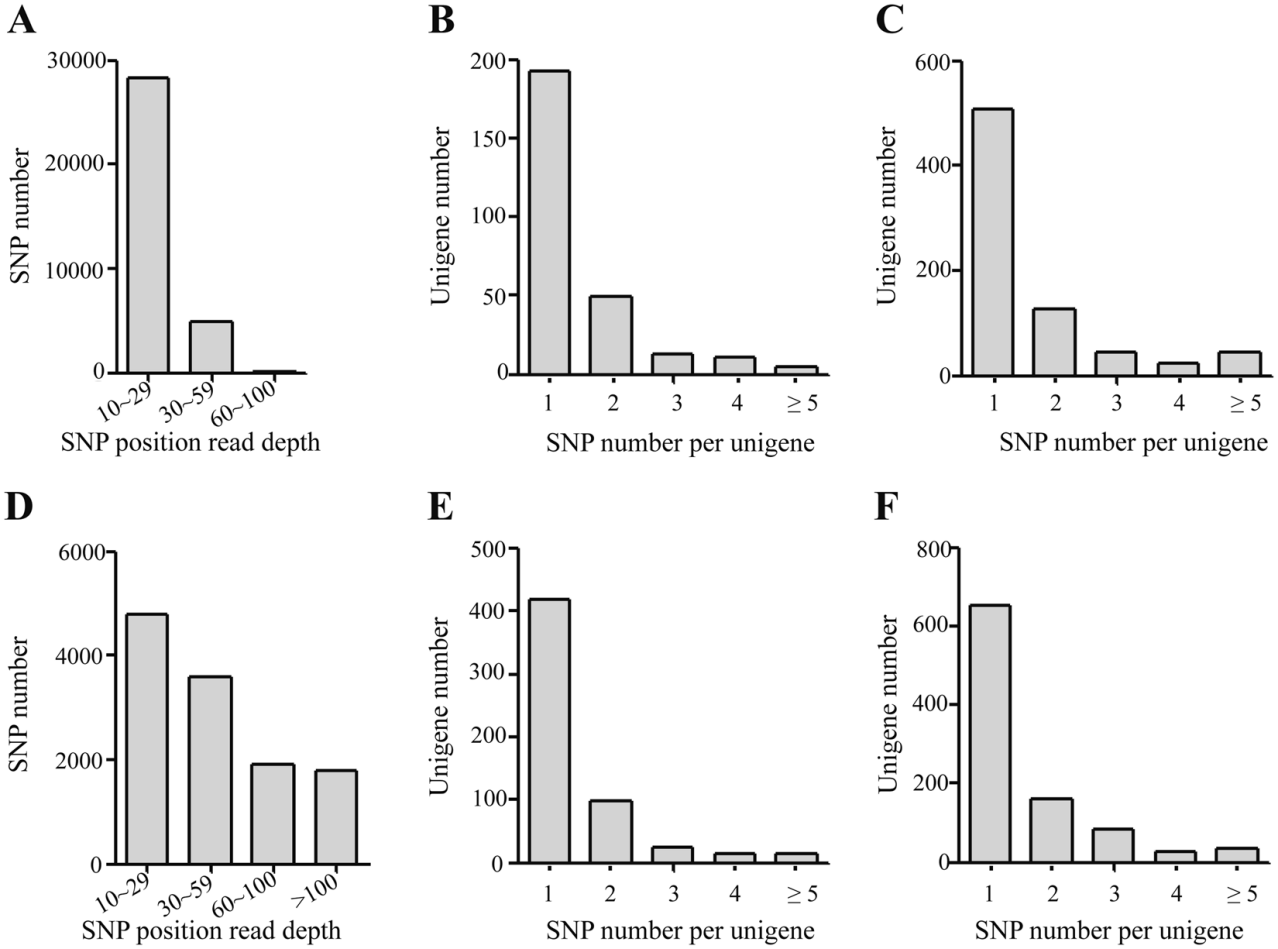
Read depth and distribution of SNPs. (**A**) and (**D**) represent read depths at SNP positions in transcriptomes in individuals and cell lines, respectively. (**B**) and (**C**) stand for SNP distribution in resistant and susceptible individuals, respectively. (**E**) and (**F**) represent SNP distribution in resistant and susceptible cell lines, respectively.

As the read depth in SNP position is closely related to the prediction accuracy of SNP^18^, statistical analyses of read depth for each SNP were performed in the transcriptomes of individuals (including SS1, SR2, KS3 and KR4 libraries) and cell lines (including C1, R2 and S3 libraries), respectively (Fig. 2A and D). SNPs with a read depth between 10 and 59 times account for 99% and 67% in individuals and cell lines respectively. While SNPs with a read depth from 60 to 100 times and more than 100 times account for nearly 16% in cell lines separately. Among these SNPs associated with resistance/susceptibility to GCRV, corresponding genes with single SNP were more common and those with no more than 5 SNPs occupied more than 94% of total genes in individuals and cell lines (Fig. 2B,C,E,F and Supplementary Dataset 4). On the other hand, the number of genes with single SNP was nearly 2.6-fold higher in susceptible groups than that in resistant groups in individuals (Fig. 2B and C) and approximately1.5-fold in cell lines (Fig. 2E and F).

**Figure 3:**
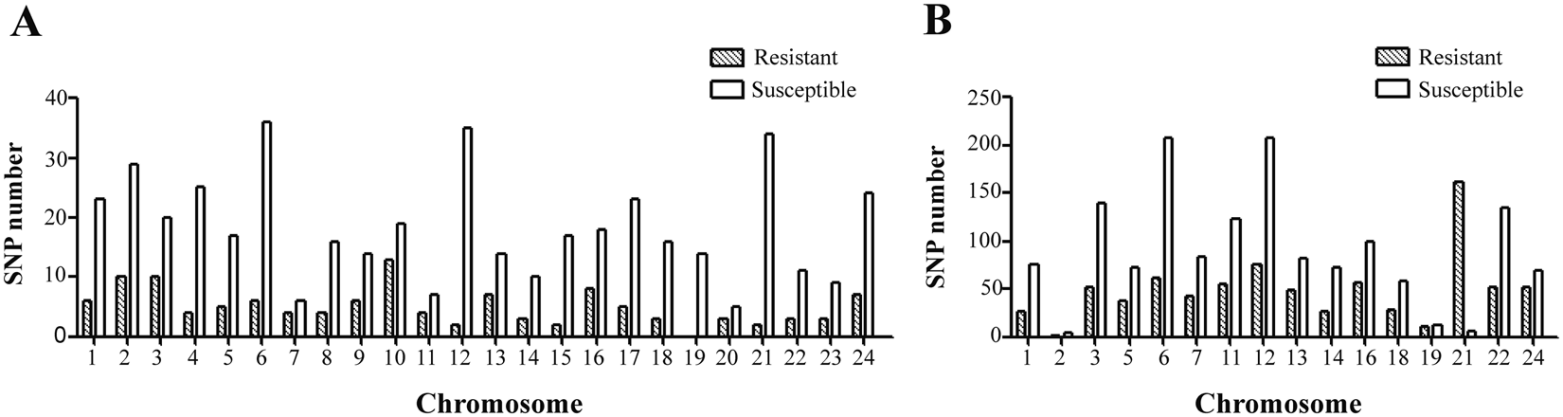
SNPs location in *C. idella* genome. 24 *C. idella* chromosomes are marked on the horizontal axis and the number of SNPs associated with resistance/susceptibility to GCRV is plotted on the vertical axis in individuals (**A**) and cell lines (**B**), respectively.

SNP distribution among genes is important when considering the marker density and genome coverage^19^. We examined the genomic distribution of SNPs associated with resistance/susceptibility to GCRV by BLAST analysis and found that SNPs from individuals were located throughout the genome (Fig. 3A). However, no SNP was blasted to the chromosomes 4, 8, 9, 10, 15, 17, 20 and 23 in cell lines (Fig. 3B). The distribution of SNPs in susceptible samples was more dispersed than that in resistant samples of individuals and cell lines (excluding the SNPs that could not be mapped to genome) (Fig. 3 and Supplementary Dataset 5). The number of SNPs in susceptible groups was 2-fold higher than that in resistant groups except on chromosomes 7, 10, 11 and 20 in individuals and chromosomes 5, 7, 13, 16, 19, 21 and 24 in cell lines. More than 30 SNPs were located on chromosomes 6, 12 and 21 in susceptible group in individuals (Fig. 3A), and more than 200 SNPs were located on chromosomes 6 and 12 in susceptible group in cell lines (Fig. 3B). On the contrary, chromosome 21 carried more than 150 SNPs associated with resistance to GCRV, which was 10-fold higher than those in susceptible groups in cell lines (Fig. 3B).

### Gene annotation and functional analysis

**Figure 4:**
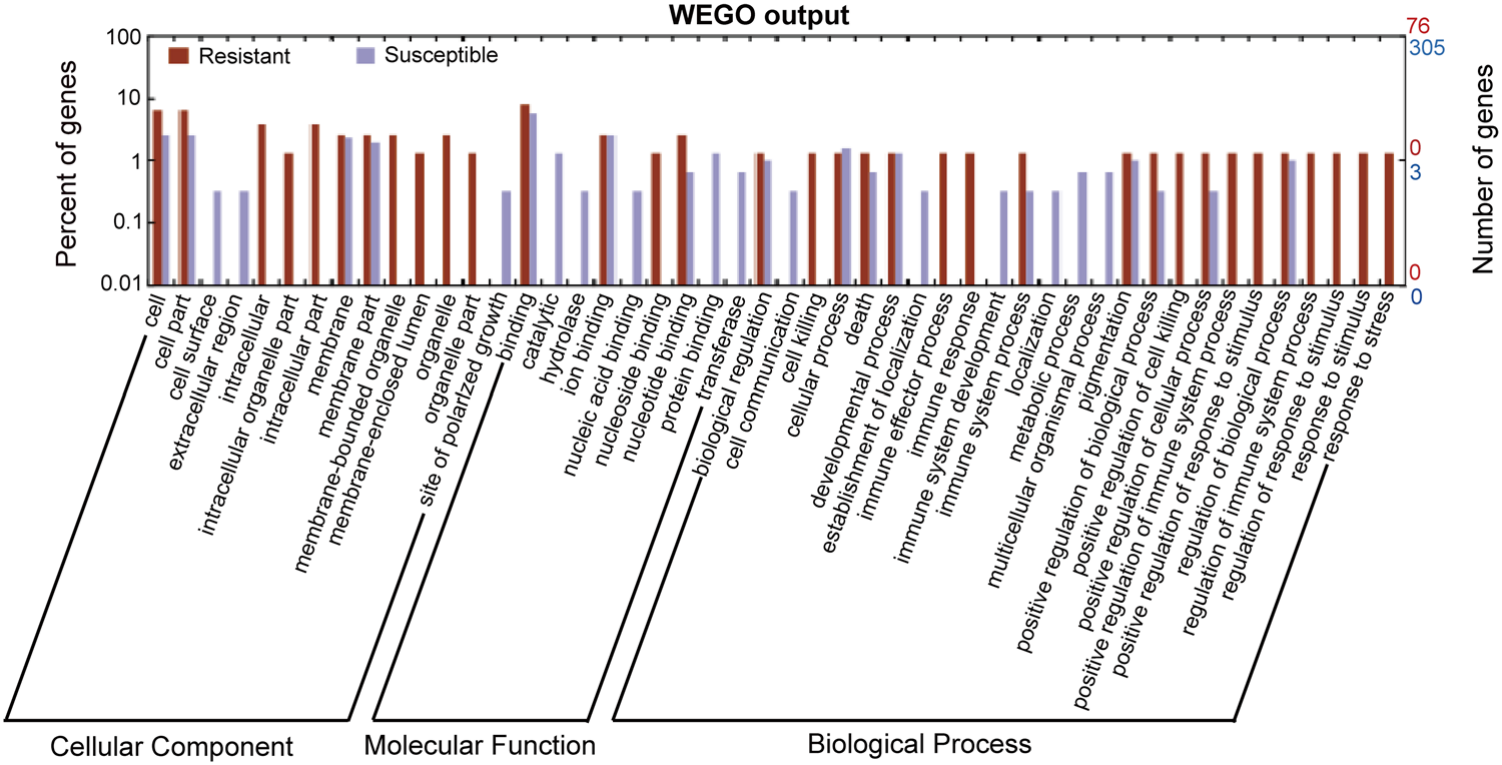
GO analysis of the annotated genes with resistance/susceptibility-associated SNPs in individuals. Red columns represent GO terms of genes containing resistance-associated SNPs and blue columns stand for GO terms of genes containing susceptibility-associated SNPs. And vertical axes show the number and percentage of corresponding genes respectively.

Gene ontology (GO) categories and kyoto encyclopedia of genes and genomes (KEGG) pathway analyses were performed to annotate genes containing resistant/susceptible SNPs in individuals. Totally, 1,024 transcripts (1,794 SNPs) consist of 272 resistant transcripts (413 SNPs) and 752 susceptible transcripts (1,381 SNPs), in which, 129 resistant transcripts (192 SNPs) and 401 susceptible transcripts (783 SNPs) can be blasted to the corresponding genes according to the genome annotation of *C. idella* (Supplementary Dataset 3). All transcripts had significant hits to proteins in the non-redundant database and these genes were annotated by the corresponding top best BLASTX hit. After GO annotation, many genes were assigned with one or more GO terms. The plotted GO annotation of these genes are shown in Fig. 4. The number of genes with a GO term was 76 and 305 in resistant and susceptible groups respectively (Supplementary Dataset 6).

**Figure 5:**
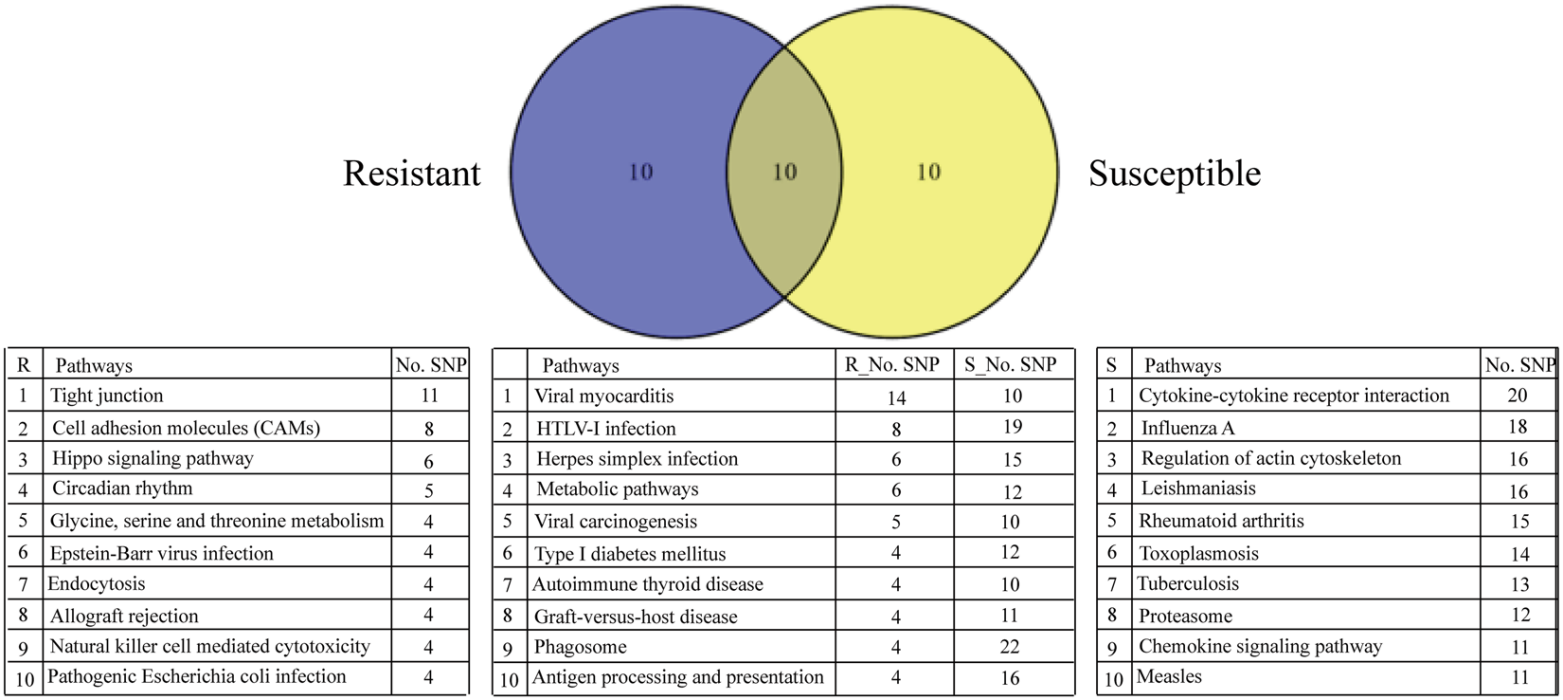
Top 20 KEGG pathways of genes containing SNPs associated with resistance/susceptibility to GCRV in individuals. Venn diagram describes the overlapped pathways between resistant and susceptible groups. The number of SNPs is shown behind the corresponding pathways.

In addition, the top 20 assignments to KEGG pathways in resistant and susceptible groups are shown in Fig. 5 and Supplementary Dataset 7. The venn diagram describes the overlap between resistant and susceptible top 20 assignments. Herein, 10 pathways were shared between resistant and susceptible groups. In these pathways, the number of SNPs in susceptible groups is 2-fold higher than that in resistant groups except viral myocarditis pathway. The pathway with the highest density of SNPs in resistant groups is viral myocarditis with 14 SNPs, and that in susceptible groups is phagosome with 22 SNPs (Fig. 5).

### SNP verification and significant relationship with resistance/susceptibility to GCRV

**Figure 6:**
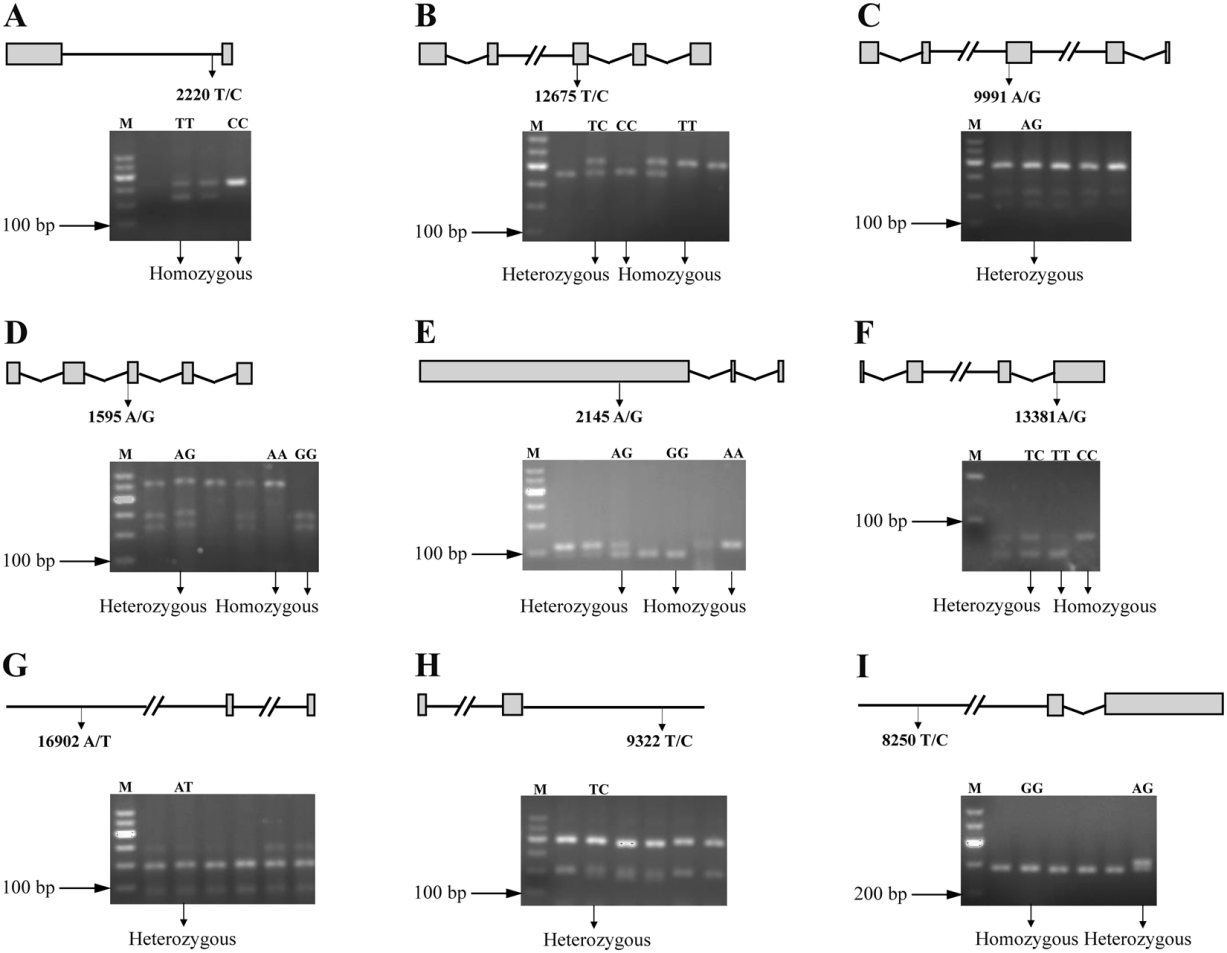
Gene structure schematics and polymorphism site confirmation. 22 SNPs associated with resistance/susceptibility to GCRV in individuals were selected for SNP verification, and 9 SNPs were confirmed. (**A–I**) show the 9 proved SNPs, including *Hnrpa*, *Yes*, *Zfyve26*, *Gsto*, *C7N1*, *Napi-llb2*, *Mef2d*, *Hiat* and *Chsg* genes, respectively. Double slash, grey box and polygonal line represent ellipsis, exons and introns, respectively. The polymorphism sites are marked by vertical arrows in gene organization plots and the genotypes of SNPs are demonstrated on the corresponding electropherogram. M, Maker 1.

During the whole challenge experiment, no dead fish was found in the control group. According to the symptoms and death status, 30 fish were grouped into resistant stock and 30 fish were grouped into susceptible stock. Based on the results of above venn diagram analysis (Fig. 1G), 22 genes with single SNP associated with resistance/susceptibility to GCRV were selected to validate their reliability. The primers for SNP verification were listed in Table 2. The results showed that 9 SNPs were validated by PCR-RFLP and their target fragment can be digested with corresponding enzyme, their gene structure and polymorphism confirmation were displayed in Fig. 6. Agarose gel electrophoresis showed that *Gsto*, *Yes*, *C7N1* and *Napi-llb2* genes contain three genotypes, *Hnrpa* and *Chsg* have two genotypes, while *Hiat*, *Mef2d* and *Zfyve26* are heterozygote and possess just one genotype (Fig. 6). Subsequently, SNPs with more than one genotype were genotyped in resistant/susceptible groups and the results were listed in Table 3. Genotyping and association analysis results preliminarily revealed that heterozygote in the site (4807309 A/G) of *C7N1* gene was significantly more in resistant population than that in susceptible population (*P* = 0.017). Aside from this SNP, there was no other SNP that was significantly related to the resistance/susceptibility to GCRV (*P* > 0.05) (Table 3).

**Table 2:** Primers used in the experiment.

Gene	Primer name	Forward primer (5′-3′)	Primer name	Reverse primer (5′-3′)	Temperature (°C)
CI01000000_08975466_08977875	HNOF658	GGTATGTAATCAACCTGTCTTCA	HNOR659	GTTGATGTGGGCAGAGTCC	54.5
	HNIF660	ATTTAAAGGTTTTTTTTTTTTTGT	HNIR661	GTTAAAGTTAAGAAAAAAAGAAAGAA	54.5
CI01000012_12132327_12133572	GSF582	TCTGCTTGTAGAAACTGCTGAT	GSR583	CATTGTGACTGAATCGCCT	54.1
CI01000020_05320359_05335741	CRF673	CCATCCCACCCGCTTCA	CRR674	ATCGCTGTCGCCTCGGA	60.0
CI01000000_15829395_15841876	AHF719	GATTTCCATGCCAGATGTTGTCT	AHR720	TCTACTTCTGGTGCTTTAATGTCTCC	60.0
CI01000001_04462781_04463560	FBF588	TGAATGACAAGATGGTGAGCAA	FBR589	GTGAGTGAAACGAATAAACCGAA	58.9
CI01000001_05575469_05577709	HYF715	ATAAAGCAGCACCAAAAGG	HYR716	TTCATTAATGTAATGAAGATCTCAC	53.2
CI01000001_10604980_10633286	MLCF596	CGAGTCAGTGGATGGAAAAG	MLCR597	GGTTATGGATGGGTTAGAGACT	54.6
CI01000004_11432849_11451784	YEF586	TTTCCGTCTGAGATTGTTTGTT	YER587	ACCAGATTGTCTCCCACCAG	56.7
CI01000004_15217903_15228632	ELF679	TTTAGATTGATTTCTGAAGGAT	ELR680	TTTTACAATTGTCTACTTGAATATA	50.9
CI01000004_15326982_15360355	SKF600	GCATCCTGCCTGTCAACG	SKR601	AGTTTGCTGTTGGGGTCG	56.8
CI01000006_12684154_12695960	DNF711	CAACAACGAGGACACCCC	DNR712	TTTCTTCCCTTTTGTCTCTGC	56.0
CI01000012_07509419_07535325	ZFF723	TGGCTCTCCCAGTGCTCTA	ZFR724	TTGCTGACGGAGGTTCTGT	56.0
CI01000013_04750261_04754885	DEF592	GATGCCAGTTCACTCTTTG	DER593	ACACTGATGACGAAACTCTATT	50.5
CI01000013_04805165_04810980	C7NF580	GACTGAGAATGCTGTGAAAGATG	C7NR581	CAATGAGGTGGGATTTTAGTGA	56.7
CI01000016_04557546_04579200	ERF594	CCCTTCTTCGTCCCTCCTA	ERR595	CGGAGCTTCAGTGGGATAC	55.8
CI01000016_05878037_05885761	FLF702	AAATTTCCTCTTTGAACTTTTT	FLR703	CTTTACCTCTGCCATCCAC	52.5
CI01000021_00349073_00352551	ZHF654	AGATTGTTGTTCTGGTGCCTGA	ZHR655	ACGGTGGACTTCCTCTTGCTA	59.0
CI01000021_06435903_06449790	NAF700	CACTTCTTTTTCCACATCTG	NAR701	TTTGGCCAATCGGATAG	50.3
CI01000024_01747187_01758168	INF721	CGATATCATTATAAATTGAATGATG	INR722	CAGTATGCCTGTTGATAAAAGC	54.5
CI01000027_07709714_07744049	MEF675	TGTTTCATTAGGAGTCGGATT	MER676	CGAGAGATAAACAAGTCCAAAG	54.1
CI01000030_07749038_07754607	HIF584	CAGCGTGTTCCCCTTATCAG	HIR585	CAAAGTCTGAAACTGAACTCGGT	58.1
CI01000036_01250575_01252253	CHF713	GCTTTCTTAATGTGCCCGTCT	CHR714	TCCTCCTCACCATCATTCCC	59.0
CI01000000_08975466_08977875	HF731	ACTGCGTGGTGGTCCAAAAC	HR732	GCCGAACTGCGAGAAATAATC	60.0
CI01000004_11432849_11451784^*^	YF735	ACAACTTCAACAGCCGCACA	YR736	AGGGAAGGGGTTGCTCACA	60.0
CI01000012_12132327_12133572^*^	GF744	GAAGAATCCTTTTGGGACGGTA	GR745	TTCTCAGGGTAGACCTCATCCAG	60.0
CI01000013_04805165_04810980^*^	C7F739	AAATCCCACCTCATTGCCTTA	C7R740	TGAAGACGGGCTTGTTTGC	60.0
CI01000021_06435903_06449790^*^	NF741	GAACCAAACGGAAACCATCAA	NR742	CAACAAAATCAACCCCACAGC	60.0
CI01000036_01250575_01252253^*^	CF737	CAGCGAGAATGCCATCTTGAC	CR738	GGGATTTTGACCGTAGGATAGC	60.0

Note: *Indicates the primers used in the qRT-PCR, other primers are used in the SNPs verification.

**Table 3:** Distribution of the SNPs in resistant and susceptible groups.

Gene	Locus	Genotype	Resistant	Susceptible	*χ* ^2^ (*P*)	Allele	Resistant	Susceptible	OR (95% CI)	*χ* ^2^ (*P*)
			NO (%)	NO (%)			NO (%)	NO (%)		
Hnrpa	8975656 T/C	TT	27 (90.0)	30 (100.0)	3.157 (0.075)	T	54 (90.0)	60 (100.0)	NA	6.315 (0.011^*^)
		TC	0 (0.0)	0 (0.0)		C	6 (10.0)	0 (0.0)		
		CC	3 (10.0)	0 (0.0)		**HWE**	**7.06E** ^**−7**^	**1.000**		
Yes	11446782 A/G	AA	3 (10.0)	5 (16.7)	1.731 (0.421)	A	25 (41.7)	24 (40.0)	1.071 (0.517–2.219)	0.034 (0.853)
		AG	19 (63.3)	14 (46.7)		G	35 (58.3)	36 (60.0)		
		GG	8 (26.7)	11 (36.7)		**HWE**	**0.097**	**0.879**		
Gsto	12133107 A/G	AA	7 (23.3)	7 (23.3)	0.138 (0.933)	A	33 (55.0)	32 (53.3)	1.069	0.034 (0.855)
		AG	19 (63.3)	18 (60.0)		G	27 (45.0)	28 (46.7)		
		GG	4 (13.3)	5 (16.7)		**HWE**	**0.126**	**0.261**		
C7N1	4807309 A/G	AA	3 (10.0)	4 (13.3)	8.174 (0.017^*^)	A	30 (50.0)	22 (36.7)	1.727 (0.833–3.582)	2.172 (0.141)
		AG	24 (80.0)	14 (46.7)		G	30 (50.0)	38 (63.3)		
		GG	3 (10.0)	12 (40.0)		**HWE**	**0.001**	**0.979**		
Napi-llb2	6436410 A/G	AA	1 (3.3)	1 (3.3)	5.612 (0.060)	A	11 (18.3)	20 (33.3)	0.448 (0.192–1.046)	3.523 (0.060)
		AG	9 (30.0)	18 (60.0)		G	49 (81.7)	40 (66.7)		
		GG	20 (66.7)	11 (36.7)		**HWE**	**0.999**	**0.159**		
Chsg	1260503 A/G	AA	0 (0.0)	0 (0.0)	0.077 (0.781)	A	10 (17.2)	9 (15.0)	1.133 (0.424–3.023)	0.062 (0.802)
		AG	10 (33.3)	9 (30.0)		G	48 (82.8)	51 (85.0)		
		GG	20 (66.7)	21 (70.0)		**HWE**	**0.544**	**0.610**		

Note: ^*^The distributions of corresponding SNPs between resistant and susceptible groups are significantly different (*P* < 0.05). ‘NA’ indicates not available. *P* values for HWE test are shown with boldface.

The mortality of *C. idella* post GCRV infection ranges from 50% to 90%, and that of rare minnow (*Gobiocypris rarus*) is almost 100%. Interestingly, *D. rerio* post GCRV challenge is nearly 100% survival^20^. In order to investigate the differences among them, comparisons of genomic base types of the corresponding SNP positions among *C. idella*, *G. rarus* and *D. rerio* were listed in Supplementary Table S2. Meanwhile, chromosome locations of 22 SNPs and corresponding amino acids in *C. idella* were listed in Supplementary Table S3. Most SNPs were synonymous except for the mutation of *C7N1*, which can cause a change of amino acid (M/I). *C7N1* is a novel gene that is homologous with human *C15orf39* and locates on chromosome 7 (named as *C7N1*). Interestingly, comparative genomic analysis revealed that corresponding base of *C7N1* SNP in *G. rarus* and *D. rerio* were G and A, respectively (Supplementary Table S3). Polymorphisms in resistant and susceptible groups were in Hardy-Weinberg equilibrium (HWE) (Table 3). These results threw light on the significant association with resistance/susceptibility to GCRV. Furthermore, considering some SNPs may change the modifications (phosphorylation and ubiquitination) and localizations of proteins which are very critical for protein functions, and SNPs could change transcription and translation levels of genes by interacting with microRNAs, corresponding predictions were conducted (http://www.expasy.org/tools/). But these results revealed that there were no differences among them.

### Gene expression signatures in spleen tissue and *C. idella* kidney (CIK) cells

**Figure 7:**
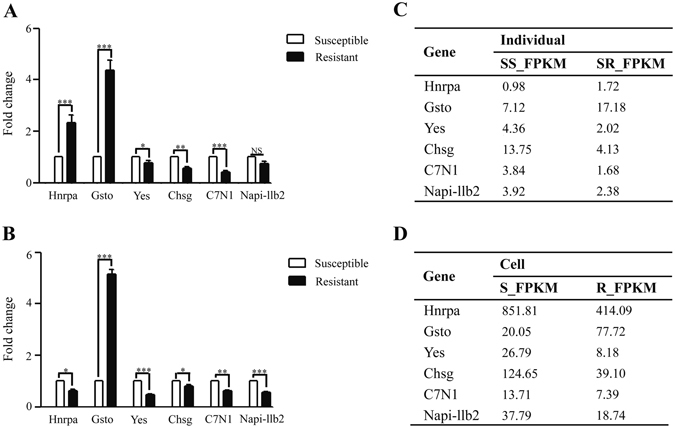
mRNA expression profiles in spleen tissue and CIK cells. Transcripts of 6 genes with more than one genotype in 9 genes with confirmed SNPs were quantified in resistant and susceptible groups in spleen (**A**) and CIK (**B**) by qRT-PCR (n = 3). *18 S rRNA* and *EF1α* as reference genes were used in individuals and cells respectively. Fold changes of mRNA expressions were relative to corresponding susceptible samples (defined as 1). *P < 0.05; **P < 0.01; ***P < 0.001; ‘NS’, not significant, *P* > 0.05. The normalized absolute quantification FPKM values of corresponding genes in RNA-Seq datasets of spleen and CIK were demonstrated in (**C**) and (**D**), respectively.

Intraspecific allelic variation may bring phenotypic variation by influencing the gene expression, including the possibility of hybrid vigour as beneficial traits that are exploited in animal and crop breeding^2, 21^. To investigate mRNA expression levels of genes corresponding to resistant/susceptible SNPs, we selected 6 genes with validated genetic variation to examine their mRNA expression levels in spleen tissue and CIK cells. The results showed that their expression patterns were accordant between RNA-Seq and qRT-PCR. Meanwhile, mRNA expression profiles were similar between individuals and cell lines except for *Hnrpa* (Fig. 7A and B). The expression level of *Hnrpa* was 2-fold higher in resistant groups than that in susceptible groups and was consistent with transcriptome data in individuals (Fig. 7A and C), while that was nearly 2-fold higher in susceptible groups than that in resistant groups and was consistent with transcriptome data in cell lines (Fig. 7B and D). *Yes*, *Chsg*, *C7N1* and *Napi-llb2* were highly expressed in susceptible individuals and cell lines. *Yes* and *Napi-llb2* expressions were nearly 2-fold higher in susceptible groups than those in resistant groups in cell lines, and they were 3-fold higher and 2-fold higher in susceptible groups than those in resistant groups in transcriptome of cell lines respectively (Fig. 7B and D). The expression level of *Gsto* was 4-fold higher and 5-fold higher in resistant groups than that in susceptible groups in individuals and cell lines respectively (Fig. 7A and B).

## Discussion

In recent several years, the rapid development of high-throughput sequencing technology boosts the deep and efficient probing of transcriptomes and genomes^22, 23^. It ensures a sufficient resource for SNP discovery. SNP diversity is important source for genetic diversity, molecular evolution and disease resistance. Some researchers primarily focus on non-synonymous coding SNPs, because those SNPs might influence the protein activity directly. However, human GWAS show that the synonymous SNPs play important roles as well as non-synonymous coding SNPs^24^. In this study, individuals and cell lines were challenged with GCRV and divided into resistant and susceptible groups according to their antiviral ability, and their mRNAs were sequenced to identify the different SNPs. In order to generate more SNP information associated with resistance/susceptibility to GCRV, integrative analysis was employed to explore the conserved SNPs. Herein, the purpose of this study is to develop a great many of convinced and specific SNPs for the selective breeding of GCRV resistant varieties through transcriptomes in individuals and cell lines.

It is notable that substitution is conserved in the process of evolution^25^. The ratio of transition to transversion was about 2.0 in transcriptomes of individuals and cell lines, similar to the results of other SNP studies^26^. Meanwhile, the ratio of A/T to G/C were about 7 and 6 in individuals and cell lines separately, which are similar with that in higher vertebrates, for example, it is about 5 in human^27^. These results may indicate that the preferences of base mutations result from their molecular structures and are conserved. The significant differences of SNPs and InDels in individuals (between female and male) have been reported in the *C. idella* genome^1^. However, SNPs and InDels associated with resistance/susceptibility to GCRV were not reported by omics sequencing till now. Population differentiation has also been observed in *Streblospio benedicti* ^28^. Abundant genetic diversity in wild *C. idella* germplasm resources is observed and the breeding of improved *C. idella* varieties has a good genetic basis^2^.

Owing to the read depth play important roles in prediction accuracy of SNPs^18^. One advantage of Illumina sequencing platform is the higher read depth comparing to 454 sequencing platform^29^, which could ensure that most of expected SNPs in the sequenced population could be detected^30^. It’s worth noting that SNPs with much higher read depth should be excluded since too high read depth might be caused by paralogous sequence variants^31^. In this study, read depth of SNP positions between 10 and 59 accounted for majority, meanwhile, all read depth did not surpass 300. These were enough to ensure the accuracy of predicted SNPs. What’s more, genes with single SNP were more common, which increases the coverage of SNPs associated with resistance/susceptibility to GCRV in *C. idella* genome. In view of the whole genome assembly, the exact assessment pattern of the SNP distribution in the genome is possible. For this reason, when these genes containing SNPs associated with resistance/susceptibility to GCRV were plotted to the *C. idella* genome by BLAST analysis, they had a good coverage of all 24 chromosomes. Some SNPs were not plotted to the corresponding chromosomes due to the gaps in genome sequence, which is acceptable at the genomic scale. These sequences and SNPs will provide novel materials for genome sequence amendment and facilitate the studies on selective breeding and immune mechanisms.

In this study, we identified many differential genes between resistant and susceptible population in individuals. To better understand the molecular functions of these candidate genes, we performed GO classification and KEGG pathway analyses. The ratio of differentially expressed sequences to the total sequences of corresponding GO categories or KEGG pathways were regarded as the major criteria for enrichment assessment^32, 33^. GO subcategories or KEGG pathways with high ratio of SNPs are the major concerns. Our results revealed that GO subcategory with the highest SNP enrichment ratio was “binding” in resistant and susceptible groups. GO analysis also showed that some cellular components tended to be less polymorphic. Whereas KEGG pathway analysis showed that some pathways tended to be more polymorphic. Because disease-related mutations are unequally distributed throughout protein sequences, having a higher occurrence in structurally/functionally important sites, we can expect the number of localization mutations to be higher than that of the calculated. Localization mutations are rare events, but they should be taken into account when predicting consequences of mutations^34^. Biological systems are defined by their components and the interactions among these components. Likewise, mutations can affect the components and their interactions. Mutations that alter interactions are most likely to be detrimental^35, 36^. These observations should be explored and verified in future studies.

A higher number of short-read alignments at regions of interest may be helpful in more precisely resolving the real allele frequency of mutant allele in bulked DNA^37^. Therefore, genotyping-by-sequencing (GBS) may be employed for further gene fine-mapping and allele analysis^38^. All the high-quality sequence reads were aligned to the *C. idella* whole genome^1^, with the objective of SNP identification. In this work, we picked up 22 SNPs associated with resistance/susceptibility to GCRV infection for verification and 9 SNPs were validated by PCR-RFLP. With SHEsis genotyping and association analysis in 30 resistant and 30 susceptible individuals, the mutation (4807309 A/G) in *C7N1* gene was significantly associated with resistance/susceptibility to GCRV infection in individuals. Our study preliminarily showed that heterozygous genotype A/G was significantly resistant to GCRV infection than homozygous genotype A/A and G/G (*P* < 0.05). Furthermore, comparative genomic analysis showed that the mutation site (4807309 A/G) in *C7N1* gene corresponds to A and G in *D. rerio* (resistant to GCRV) and *G. rarus* (susceptible to GCRV) respectively. These results may suggest that the base G was associated with susceptibility to GCRV and the heterozygous genotype can improve the ability of resistant to GCRV in *C. idella*. Similarly, heterozygous SNP variation can contribute to increase latex yield in the hybrid^21^. The molecular functions of *C7N1* have not been studied in fish and even higher vertebrates. The relationship between above significant SNPs and resistance/susceptibility to GCRV was preliminarily verified by an independent infection experiment, these significant SNPs can be considered as candidate genetic markers and further verification needs to be done in other natural populations in the future. In the present study, SNPs were identified at the antiviral transcriptome level, which will enable us to further understand the roles of SNPs in antiviral responses.

A powerful feature of transcriptome is fully examining changes of gene expression levels among individuals or populations. There has long been a realization that gene expression differences play vital roles in species differentiation and population adaptation^39^. Several studies on closely related species indicate that there is a genetic basis for differences in transcript levels^40^, which could lead to adaptive divergence in the wild^41, 42^. In this study, *Hnrpa* has different expression patterns between individuals and cell lines, which may indicate the difference in antiviral immune mechanism between *in vivo* and *in vitro*. Quantitative estimate of gene expression can be associated with change in nucleotide sequence^43^. Most SNPs controlling gene expression occur outside the coding regions of genes, so finding relationship between SNP genotype and expression level can provide an indirect link between SNP and phenotype^44^. Other methods can furthermore be used to study regulatory network changes by analysing co-expression patterns and associating with nucleotide changes and phenotypic traits^45^. The superiority of this approach is that experiments on natural selection for gene expression differences can now be monitored in a more effortless way than that in the past. Finally, cross-species comparisons of transcriptomes have recently shown promise for conservation genetics of endangered animals^46^ and also for enhanced understanding of the fundamental principles of population genomics^47^, allowing us to potentially predict the responses of natural populations to future environmental perturbations.

SNP as molecular marker plays vital roles in animal breeding. As an important economic fish with long growing and breeding cycle, conventional breeding in *C. idella* is labor-intensive and time-consuming^2, 48^. Therefore, it is necessary for breeders to use an effective selection method to increase breeding efficiency as opposed to the traditional pure phenotype-based selection process. Natural germplasm resources of wild *C. idella* were genetically diverse, which provides the basis for constructing basal populations for QTL and GWAS analysis^2^. This study is an important step towards the generation of SNPs in specific feature and provides precious SNP resource for the selective breeding of resistant varieties. In addition, our surveys provide valuable information that will facilitate the studies on genomic variation underlying traits of interest in fish, including immune responses, regulatory mechanisms and environmental adaptability.

## Methods

### Statement

All experiments were approved by the Animal Care Committee of Huazhong Agricultural University. The Administration of Affairs Concerning Animal Experimentation Guidelines stated approval from the Science and Technology Bureau of China. The methods were carried out in accordance with the approved guidelines. Total 200 experimental individuals were fed in four 300-liter aquaria with suitable illumination, water temperature, dissolved oxygen content, and adequate forage in the Huazhong Agricultural University, China. Approval from the Department of Wildlife Administration is not required for the experiments conducted in this paper. All surgery was performed to minimize suffering by using 3-Aminobenzoic acid ethyl ester methanesulfonate (MS-222) anesthesia.

### Transcriptome data collection

For the viral challenge, healthy *C. idella* were infected with GCRV (097 strain, suspended in phosphate-buffered saline (PBS)). In this process, the moribund fish with hemorrhagic symptom between 24 and 72 hours post challenge were regard as susceptible individuals and the surviving fish after 10 days post challenge were resistant individuals. The spleen and head-kidney tissues from 12 resistant and 12 susceptible individuals were collected and their RNA-Seq libraries were constructed and sequenced, which were divided into 4 groups: spleen tissue from susceptible fish (SS1) and resistant fish (SR2), head-kidney tissue from susceptible fish (KS3) and resistant fish (KR4). These transcriptome data were deposited in the NCBI with the Sequence Read Archive (SRA) accession number of SRP049081^8^. Meanwhile, to obtain monoclonal cells, CIK cells were digested and then filtered twice with a 150 μM nylon mesh. 100 μL Dulbecco’s modified Eagle’s medium (DMEM) supplemented with 7% fetal bovine serum (FBS), 100 U/mL of penicillin and streptomycin sulfate as well as 5 μL/mL insulin (Gibco, USA) were added to each well of the 96-well culture plates beforehand, the single cell was ultimately instilled utilizing the BD FACSAria™ III Cell Sorter (USA) and subcultured up to being the monoclonal cells with a cell density of 5 × 10^4^/well in 96-well plates. Identified as resistant, susceptible or ambiguous by three strategies: (1) CPE analysis. (2) Cell proliferation assay. (3) Antiviral activity assay. These samples were divided into control (C1, unsorted), resistant (R2) and susceptible (S3) groups. The RNA-Seq data were obtained by Illumina HiSeq2500 sequencing technology and deposited in the NCBI Gene Expression Omnibus with accession number of GSE87414. All reads were filtered with NGS QC Toolkit for further analysis.

### SNP and InDel detection

Short reads of two transcriptomes were separately mapped to *C. idella* genome using BWA version 0.5.9 (http://bio-bwa.sourceforge.net/) with the default settings except for no gap tolerance. The data from individuals (SS1, SR2, KS3 and KR4) and cell lines (C1, R2 and S3) were aligned for SNP and InDel identification. One reliable and frequently used software program, SAMtools, was independently applied for the identification of SNP and InDel with *C. idella* genome as reference sequences. InDel represents single base insertion and deletion. The software package SAMtools (http://samtools.sourceforge.net/) was used to convert sequence alignment/map (SAM) file to sort binary alignment/map (BAM) file. And the command dump was employed to remove duplicates. SNPs were further investigated by BCFtools. Those with total read depth more than 20 and mutation read depth over 10 were identified as high quality SNPs or InDels. Since accuracy of SNP prediction is dependent on sequence coverage^30^, we combined two transcriptomes to improve the prediction accuracy.

### Statistical analysis of SNP information

The ratio of mapped reads in each dataset was calculated by flagstaff command in SAMtools software^8^. SNP and InDel ratios were obtained by each type of DNA substitution and SNP read depth in the result file in SAMtools. SNPs involved in resistance/susceptibility to GCRV were analysed using the tool (venny 2.1) of BioinfoGP (http://bioinfogp.cnb.csic.es/index.html) in individuals and cell lines, respectively. Meanwhile, the number of these SNPs in genes (including exon, intron and flanking region) was analysed. And the BLAST was employed to analyse the chromosome location of genes with resistance/susceptibility SNP in individuals and cell lines.

### Functional annotation of genes containing SNPs

The genes possessing SNPs associated with resistance/susceptibility to GCRV were annotated using NCBI non-redundant database by BLASTX (e-value < 0.00001)^49^. After that, BLAST2GO software were employed to allot the genes with GO terms of biological process, molecular function and cellular component^33^. Subsequently, annotated information was imported into BGI WEGO program (http://wego.genomics.org.cn) in WEGO native format to plot GO annotation results. KEGG pathways were assigned to genes containing SNP associated with resistance/susceptibility to GCRV by the online KEGG Automatic Annotation Server (KAAS) (http://www.genome.jp/tools/kaas/). KEGG Orthology (KO) assignment was applied by Bi-directional Best Hit (BBH) method.

### GCRV infection, SNP verification, genotyping and association analysis

For verification experiment, grass carp (approximate body length of 10 cm) were collected from three fish farms (Hubei province, China) where no hemorrhagic disease of *C. idella* was found in recent years, which were different population from RNA-Seq. Fish were injected with GCRV (097 strain, suspended in PBS) at a dose of 3.63 × 10^7^ TCID_50_/g as previous report^6^. The moribund fish were sampled between 24 h to 72 h post injection and the surviving fish after 10 days post-injection were serenely sacrificed. Spleen tissue was collected and kept at −80 °C until DNA and RNA isolation. These samples were divided into resistant and susceptible groups. DNA and RNA were prepared for SNP verification and gene expression analyses.

In order to validate the accuracy of SNPs screened from transcriptome, 22 SNPs associated with resistance/susceptibility to GCRV in individuals were investigated by PCR-RFLP, tetra-primer ARMS-PCR^50^ and sequencing all samples. The primers were designed to amplify the target sequence with fragment length of 100 ~300 bp containing polymorphism sites and synthesized in TsingKe Biotech (Wuhan, China) (Table 2). 30 resistant and 30 susceptible DNA samples were used as templates for SNP verification, genotyping and association analysis. 5 μl PCR products were electrophoresed on 1.0% agarose gel for quality measuring. Another 5 μl products were digested with corresponding restriction enzyme (Supplementary Table S3) according to the protocol and the mixtures were examined by electrophoresis on 2.0% or 4.0% agarose gel. The confirmed SNPs were selected to analyse their relationship with resistance/susceptibility to GCRV.

Single site analysis and haplotype analysis of SHEsisPlus online version - beta in SHEsis (http://analysis.bio-x.cn) software were employed to estimate allele and genotype frequencies and analyse their relationship with resistance/susceptibility to GCRV. The logistic regression model was performed to verify the interaction by using SPSS 16.0 software, the odds ratio (OR) and 95% confidence interval (95% CI) were calculated. Propensity covariate adjustment was also performed to verify results. *P*-value less than 0.05 was considered to be statistically significant. HWE for genotypic frequencies was evaluated by the goodness-of-fit *χ* ^2^-test for each genotyped SNP.

### Confirmation of gene expression profiles by qRT-PCR

To confirm the expression profiles of genes containing resistant/susceptible SNPs, qRT-PCR was performed using a Roche LightCycler^®^ 480 system. Total RNA were extracted from spleen tissues and CIK cells with TRIzol Reagent (TaKaRa, Japan). *18S rRNA* and *EF1α* genes were employed as reference genes in individuals and cell lines separately. qRT-PCR and data analysis were performed according to the protocol and method as described previously^51^. Fold changes of gene expression levels were relative to corresponding susceptible samples (defined as 1). *P*-value less than 0.05 was accepted as significant difference (*P < 0.05; **P < 0.01; ***P < 0.001).

## Electronic supplementary material

Supplementary tables

Dataset 1

Dataset 2

Dataset 3

Dataset 4

Dataset 5

Dataset 6

Dataset 7
