# Supplementary material for: Large-scale SNP screenings identify markers linked with GCRV resistant traits through transcriptomes of individuals and cell lines in Ctenopharyngodon idella
Source: Sci Rep. 2017 Apr 26;7:1184. doi: 10.1038/s41598-017-01338-7 (PMC5430748; doi:10.1038/s41598-017-01338-7)
Supplement: Supplementary file 1 — Supplementary tables [file 41598_2017_1338_MOESM1_ESM.doc]

**Large-scale SNP screenings identify markers linked with GCRV resistant traits** **through transcriptomes of individuals and cell lines in *Ctenopharyngodon idella***

Zhiwei Liao, Quanyuan Wan, Xueying Shang & Jianguo Su^*^

*College of Fisheries, Huazhong Agricultural University,* *Wuhan 430070, China*

^*^Corresponding author, Tel/Fax: 86-27-87282227; E-mail address: [sujianguo@mail.hzau.edu.cn](mailto:sujianguo@mail.hzau.edu.cn)

*Keywords:* Grass carp (*Ctenopharyngodon idella*); RNA-Seq; SNP; Grass carp reovirus

**Supplementary Tables**

**Table S1. Annotation of all the 22 discrepant unigenes between the resistant and susceptible groups.**

| **Resistant** | **Non-redundant annotation** | **Gene name** |
| --- | --- | --- |
| CI01000000_08975466_08977875 | Hnrpa0 protein, partial [*Danio rerio*] | Hnrpa |
| CI01000012_12132327_12133572 | glutathione S-transferase omega 1 [*Danio rerio*] | Gsto |
| CI01000020_05320359_05335741 | cryptochrome 1a [*Phreatichthys andruzzii*] | Cry1a |
| **Susceptible** |  | |
| CI01000000_15829395_15841876 | PREDICTED: neuroblast differentiation-associated protein AHNAK isoform X8 [*Danio rerio*] | AHNAK |
| CI01000001_04462781_04463560 | PREDICTED: F-box protein 44 isoform X1 [*Danio rerio*] | Fbxo44 |
| CI01000001_05575469_05577709 | 3-beta-hydroxysteroid-Delta(8),Delta(7)-isomerase [*Danio rerio*] | Hydroxysteroid |
| CI01000001_10604980_10633286 | PREDICTED: histone-lysine N-methyltransferase MLL2-like [*Maylandia zebra*] | Mll2 |
| CI01000004_11432849_11451784 | tyrosine-protein kinase yes [*Danio rerio*] | Yes |
| CI01000004_15217903_15228632 | PREDICTED: ELAV (embryonic lethal, abnormal vision, Drosophila)-like 1 (Hu antigen R) isoform X2 [*Danio rerio*] | ELAV |
| CI01000004_15326982_15360355 | PREDICTED: ski-like protein-like [*Danio rerio*] | Skil |
| CI01000006_12684154_12695960 | dnaJ homolog subfamily C member 21 [*Danio rerio*] | DNAJC21 |
| CI01000012_07509419_07535325 | PREDICTED: zinc finger FYVE domain-containing protein 26 isoform X1 [*Danio rerio*] | ZFYVE26 |
| CI01000013_04750261_04754885 | Dennd4a protein [*Danio rerio*] | DENND4A |
| CI01000013_04805165_04810980 | novel protein [*Danio rerio*] | C7N1 |
| CI01000016_04557546_04579200 | PREDICTED: ETS domain-containing transcription factor ERF [*Danio rerio*] | ERF |
| CI01000016_05878037_05885761 | PREDICTED: folliculin isoform X1 [*Danio rerio*] | Flcn |
| CI01000021_00349073_00352551 | PREDICTED: zinc fingers and homeoboxes protein 3 isoform X1 [*Danio rerio*] | ZHX3 |
| CI01000021_06435903_06449790 | Na/Pi cotransporter NaPi-IIb2 [*Cyprinus carpio*] | NaPi-llb2 |
| CI01000024_01747187_01758168 | INO80 complex subunit D-B [*Danio rerio*] | INO80 |
| CI01000027_07709714_07744049 | PREDICTED: myocyte-specific enhancer factor 2D isoform X1 [*Danio rerio*] | MEF2D |
| CI01000030_07749038_07754607 | PREDICTED: hippocampus abundant transcript 1 isoform X1 [*Danio rerio*] | Hiat1 |
| CI01000036_01250575_01252253 | PREDICTED: chondroitin sulfate glucuronyltransferase isoform X2 [*Danio rerio*] | Chsg |

**Table S2.** **Base types of the identical SNP positions among *C. idella*, *G. rarus* and *D. rerio*.**

| **Resistant** | **Position** | ***C. idella* Ref** | ***C. idella* Mut** | ***C. idella* Gen** | ***G. rarus*** | ***D. rerio*** |
| --- | --- | --- | --- | --- | --- | --- |
| CI01000000_08975466_08977875 | 8975656 | C | T | C | G | C |
| CI01000012_12132327_12133572 | 12133107 | A | G | A | G | G |
| CI01000020_05320359_05335741 | 2149834 | T | C | T | - | T |
| **Susceptible** | | | | | | |
| CI01000000_15829395_15841876 | 15832815 | G | A | A | G | A |
| CI01000001_04462781_04463560 | 4453701 | C | T | C | - | Del |
| CI01000001_05575469_05577709 | 5575304 | A | G | G | Del | Del |
| CI01000001_10604980_10633286 | 10628876 | T | C | C | - | T |
| CI01000004_11432849_11451784 | 11446782 | G | A | G | G | G |
| CI01000004_15217903_15228632 | 15223532 | G | A | G | A | A |
| CI01000004_15326982_15360355 | 15358547 | T | C | C | - | T |
| CI01000006_12684154_12695960 | 12694213 | C | T | C | C | T |
| CI01000012_07509419_07535325 | 7519441 | G | A | G | - | G |
| CI01000013_04750261_04754885 | 4735877 | G | T | G | G | G |
| CI01000013_04805165_04810980 | 4807309 | G | A | G | G | A |
| CI01000016_04557546_04579200 | 4577672 | C | T | C | - | C |
| CI01000016_05878037_05885761 | 5885473 | C | T | T | - | T |
| CI01000021_00349073_00352551 | 348929 | C | T | T | T | T |
| CI01000021_06435903_06449790 | 6436410 | A | G | A | G | A |
| CI01000024_01747187_01758168 | 1746117 | C | T | T | T | T |
| CI01000027_07709714_07744049 | 722902 | T | A | T | - | Del |
| CI01000030_07749038_07754607 | 7748198 | C | T | C | - | Del |
| CI01000036_01250575_01252253 | 1260503 | G | A | G | - | G |

Note: - indicates no corresponding homologous genes. Del indicates deletion. *C. idella Ref*, *C. idella Mut*, *C. idella Gen*, *G. rarus* and *D. rerio* represent *C. idella* RNA-Seq reference base, *C. idella* RNA-Seq mutation base, *C. idella* genome base, corresponding base in *G. rarus* and *D. rerio*, respectively.

**Table S3.** **SNPs, corresponding amino acids, chromosome localizations and restriction endonucleases for verification.**

| **Resistant** | **Base type** | **Ref** | **Mut** | **Location** | **Restriction enzyme** |
| --- | --- | --- | --- | --- | --- |
| CI01000000_08975466_08977875^*^ | T/C | - | - | Chro13 | - |
| CI01000012_12132327_12133572^*^ | A/G | K | K | Chro5 | MboI |
| CI01000020_05320359_05335741 | T | - | - | Chro11 | BglI |
| **Susceptible** | | | | | |
| CI01000000_15829395_15841876 | A | F | F | Chro13 | MboII |
| CI01000001_04462781_04463560 | T | - | - | Chro6 | HinlI |
| CI01000001_05575469_05577709 | A | - | - | Chro6 | BseDI |
| CI01000001_10604980_10633286 | T | A | A | Chro6 | MnlI |
| CI01000004_11432849_11451784^*^ | A/G | A | A | Chro22 | HaeIII |
| CI01000004_15217903_15228632 | A | - | - | Chro22 | AccI |
| CI01000004_15326982_15360355 | T | N | N | Chro22 | HincII |
| CI01000006_12684154_12695960 | G | S | S | Chro3 | PstI |
| CI01000012_07509419_07535325^*^ | A/G | E | E | Chro5 | BpuEI |
| CI01000013_04750261_04754885 | G | L | L | Chro7 | HpyF10VI |
| CI01000013_04805165_04810980^*^ | A/G | M | I | Chro7 | NlaIII |
| CI01000016_04557546_04579200 | T | Q | Q | Chro12 | BselI |
| CI01000016_05878037_05885761 | T | - | - | Chro12 | XmnI |
| CI01000021_00349073_00352551 | T | - | - | Chro6 | Bsp1286I |
| CI01000021_06435903_06449790^*^ | A/G | C | C | Chro6 | BstXI |
| CI01000024_01747187_01758168 | T | - | - | Chro18 | BccI |
| CI01000027_07709714_07744049^*^ | A/T | - | - | Chro12 | SspI |
| CI01000030_07749038_07754607^*^ | A/G | - | - | Chro24 | HinfI |
| CI01000036_01250575_01252253^*^ | A/G | - | - | Unknown | Esp3I |

Note: * indicates the confirmed SNPs; - indicates the mutations that cannot be translated into amino acid or digested by restriction enzyme.
